# Heterotropic regulation and negative homotropic cooperativity

**DOI:** 10.1002/2211-5463.70317

**Published:** 2026-07-28

**Authors:** Veronica Morea, Francesco Angelucci, Federica Arnesano, Gianmarco Pascarella, Patrizio Di Micco, Andrea Bellelli

**Affiliations:** ^1^ Institute of Molecular Biology and Pathology CNR Rome Italy; ^2^ Department of Life, Health, and Environmental Sciences University of L'Aquila L'Aquila Italy; ^3^ Department of Biochemical Sciences “A. Rossi Fanelli” Sapienza University of Rome Rome Italy; ^4^ Institute of Biomembranes, Bioenergetics and Molecular Biotechnologies CNR Bari Italy

**Keywords:** allostery, arginine repressor, D‐3‐phosphoglycerate dehydrogenase, KNF model, MWC model, uracil phosphoribosyltransferase

## Abstract

Heterotropic regulation of protein function is crucial for such diverse cellular events as transcription, signal transduction, enzymatic activity, transport and many others. It is achieved via ligand‐induced changes in protein structure and is often coupled to homotropic cooperativity. In this work, we compare the structural and functional properties of a set of evolutionarily unrelated proteins that present heterotropic regulation and negative homotropic cooperativity, for at least some of their ligands. This set is limited because the coupling of negative cooperativity and heterotropic regulation occurs rarely. Nevertheless, we identified some recurring structural features among the proteins in our dataset. All of these proteins: (i) are homo‐oligomers; (ii) have a one ligand per subunit binding stoichiometry both for their primary ligands/substrates and their effectors; (iii) do not require complete saturation with their effectors to reach maximum activation or inhibition; and (iv) show peculiar intersubunit ligand‐induced asymmetry. Together, these results imply that heterotropic regulation acts at the quaternary, as well as tertiary, structure level.

## Graphical abstract

We identified a structural module common to some proteins that couple negative cooperativity with heterotropic regulation, two features that rarely coexist. These proteins are ring‐like and present an ordered asymmetry whereby noncontacting subunits are symmetric, and their tertiary structure differs from that of contacting subunits. The implications of these findings are discussed in relation to commonly invoked allosteric models.

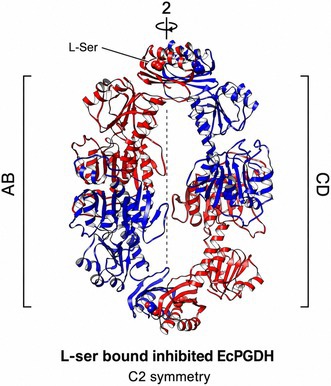

**Abbreviations**
3D: three dimensional
ArgR: arginine repressor from *E. coli*
DAHPS: 3‐deoxy‐D‐arabino‐heptulosonate‐7‐phosphate synthase
D‐LacDH: bacterial D‐lactate dehydrogenase
DPG: D‐glycerate bis‐phosphate
FBPase: Fructose bis‐phosphatase
G3PDH: Glyceraldehyde 3‐phosphate dehydrogenase
G3PDH: mammalian glyceraldehyde‐3‐phosphate dehydrogenase
KNF: cooperativity model of Koshland, Nemethy and Filmer
MWC: allosteric model of Monod, Wyman and Changeux
PGDH: EcPGDH: *E. coli* D‐3‐phosphoglycerate dehydrogenase
RMSD: root mean square deviation
UPTase: *S. solfataricus* uracil phosphoribosyltransferase
VD: allosteric model of Viratelle and Seydoux

## Introduction

The ability of even the simplest biological systems to respond to changes in the environment is key to adaptation. This phenomenon was called by J. Monod ‘the second secret of life’, the first being the transmission of genetic information by nucleic acids. The fundamental processes of the second secret of life have been largely unveiled. They mainly rely on the ability of some proteins to change their 3D structure and function upon ligand binding, thus acting as molecular switches and triggering a response at the organism level.

Regulation can result either in the enhancement or in the inhibition of protein function; the ligands that determine these effects are called positive and negative effectors, respectively. Ligands are also called orthosteric (or homotropic) and allosteric (or heterotropic) depending on whether they bind to the same site as the primary endogenous ligand (*e.g*. the substrate) or to a different site, respectively. The distinction between the ‘primary ligand’ and the ‘allosteric effector’ is based on their biological roles, and it does not entail differences at a thermodynamic level. Only in the case of enzymes, this distinction relies on a precise thermodynamic difference, given that substrates, but not effectors, may be converted to products.

Cooperativity is a sophisticated type of protein modulation that occurs in protein oligomers comprising identical or highly similar subunits, each able to bind one ligand molecule. In cooperative oligomers, ligand binding to one site/subunit can result either in the enhancement (positive cooperativity) or in the decrease (negative cooperativity) of the affinity of other identical binding sites/subunits; as a consequence, ligand affinity is regulated by the number of bound ligands. In addition to homotropic regulation, oligomers can undergo heterotropic regulation by effectors that bind to sites different from that of the principal ligand.

The mechanisms underlying (homotropic) cooperativity and heterotropic regulation have been extensively studied, and different mathematical models have been proposed to describe them (see 1, and references therein). The concerted model elaborated by Monod, Wyman and Changeux in 1965 (MWC) [1], postulates that homo‐oligomers exists in (at least) two alternative, symmetric, rapidly interconverting quaternary structures, having different ligand affinities, whose equilibrium is described by the allosteric constant L that is affected by both substrates and effectors. This model may be extended by postulating more alternative structures coexisting in a ‘conformational ensemble’ [2, 3, 4]; this is consistent with the dynamic nature of proteins, and in many cases has received experimental validation. For example haemoglobin is known to present at least three high O_2_ affinity quaternary structures, called R, R2 and R3. In the sequential model by Koshland, Neméthy and Filmer (KNF) [5], ligand binding to one subunit determines a tertiary structure change in the same subunit, which, in turn, determines a change in its interactions with adjacent subunits. From a structural point of view, there is a crucial difference between the two models. In the MWC model, the oligomer is internally symmetric in all ligation states, that is, all subunits of the oligomer have the same ligand affinity, either that observed in the fully liganded oligomer or that observed in the fully unliganded oligomer. Since in this model homotropic and heterotropic regulation occurs at the quaternary structure level, no fixed stoichiometric ratio between the effector and the protein is required. For example haemoglobin affinity for O_2_ is homotropically regulated by O_2_ and heterotropically by bezafibrate and DPG, with stoichiometry of 4, 2 and 1 per haemoglobin tetramer, respectively. In the KNF model [5], the fully liganded and fully unliganded conformations are expected to be internally symmetric, as in the MWC model; however, at variance with the MWC model, the partially liganded intermediate is necessarily asymmetric, and homotropic and heterotropic effects occur at the tertiary structure level. Therefore, the main structural difference between the MWC and KNF models is the presence of asymmetric states in the KNF model only. It may appear anachronistic to refer to the MWC and KNF model rather than to the concept of the conformational ensemble, that might encompass both; but we believe that the two older models maintain informative power as particular cases of the more general one, because they make precise and testable hypotheses on the possible distribution of the energy minima in the conformational ensemble based on their symmetry requirements.

From the functional point of view, the MWC model in its original form is compatible only with positive cooperativity, as a consequence of its symmetry requirements; however, if these are softened, one may devise a variant of the model compatible with negative cooperativity [6]. The KNF model, on the contrary, is compatible with both negative and positive cooperativities. Moreover, heterotropic regulation is frequently associated with positive cooperativity, and rarely with negative cooperativity.

Examples of certain or probable MWC‐like proteins include vertebrate haemoglobins [7, 8, 9]; Asp transcarbamoylase [10, 11]; phosphofructokinase [12]; and glycogen phosphorylase [13]; all of these show positive cooperativity. Examples of certain or probable KNF‐like proteins include *E. coli* Asp receptor [14]; bacterial D‐lactate dehydrogenase (D‐LacDH) [15]; mammalian glyceraldehyde‐3‐phosphate dehydrogenase (G3PDH) [16]; all of these show negative cooperativity (and, in the case of D‐LacDH, also positive cooperativity, depending on the ligand).

To identify whether general structural parameters related to the reaction mechanism existed, we recently analysed a small collection of proteins and enzymes that had been demonstrated to obey either a concerted or sequential reaction mechanism, and were available in the liganded, unliganded and, in some cases, also partially liganded states [17]; successively, we extended our analysis to a larger set of proteins whose reaction mechanism had not been assigned with certainty [18]. Extensive structural comparisons between either whole homo‐oligomers or their individual subunits in different ligation states showed that the concerted and sequential mechanisms are associated with different contributions of tertiary and quaternary structure changes and with different degrees of symmetry in the liganded state that may help distinguish between the two mechanisms. As expected, we found that: (i) protein homo‐oligomers that obey a concerted reaction mechanism (MWC model) present small changes in the tertiary structure of the subunits, large changes in the quaternary structure of the oligomer and highly symmetric quaternary structures in both the unliganded and liganded states; (ii) protein homo‐oligomers obeying a sequential reaction mechanism (KNF model) present relatively large changes in tertiary structure, which account for most of the overall ligand‐induced structure changes, and large intersubunit asymmetry in the partially and in the fully liganded states, although only the first, and not the latter occurrence is contemplated by the KNF model. Moreover, we found that the association of negative homotropic cooperativity with heterotropic regulation by noncovalent effectors is uncommon; indeed, only three oligomers in the extended dataset [7] present both negative homotropic cooperativity and heterotropic regulation, namely: *E. coli* D‐3‐phosphoglycerate dehydrogenase (PGDH) [19, 20, 21, 22, 23, 24]; *S. solfataricus* uracil phosphoribosyltransferase (UPTase) [25, 26, 27]; and arginine repressor (ArgR) from *E. coli* [28, 29]. In the present work we analyse the structural and functional properties of these three quite peculiar homo‐oligomers. We remark that for PGDH and ArgR essentially two structures have been described, one for the active, the other for the inhibited conformation, whereas for UPTase several different structures are available that allow one to analyse a larger fraction of the conformational ensemble of this protein.

## Materials and methods

The atomic coordinates of the proteins selected for our analysis were downloaded from the Protein Data Bank (PDB) [30].

The structural properties of the selected proteins in their liganded and unliganded state(s) were analysed using the software UCSF Chimera [31], PyMol [32] and Swiss‐PDBViewer [33]. Interface contacts were analysed using the program Face2Face [34] using default values. Accordingly, two residues are defined to be in contact if they contain atoms whose distance apart is ≤4 Å.

For the selected proteins, we performed pairwise superpositions and measured the RMSDs of equivalent α‐carbon atoms. The analysed protein regions include: (i) whole oligomers; (ii) individual subunits; (iii) intermediate oligomerization states (i.e. dimers, in the case of the PGDH and UPTase tetramers; and trimers, in the case of the ArgR hexamer). Comparison of either individual subunits or oligomers in the liganded vs. unliganded structures states were performed to assess the extent of ligand‐linked tertiary and overall (i.e. comprising both tertiary and quaternary) structural changes, respectively. Comparison of subunits in different oligomerization states and the same ligation state were performed to assess intramolecular asymmetry in the liganded and in the unliganded state.

Since RMSD values are not additive, the contribution of quaternary structure changes to overall RMSD values cannot be calculated from the RMSD values for oligomers and individual subunits. For this reason, we previously introduced the parameter R1 [7, 17], defined as the ratio between the averaged RMSD values calculated for the individual subunits in the liganded vs. unliganded state, which accounts for tertiary structure variations, divided by the RMSD values calculated for the whole homo‐oligomer in the liganded vs. unliganded state, which accounts for overall (i.e. tertiary and quaternary structure variations). Low and high R1 values indicate that overall changes are mostly contributed by quaternary and tertiary variations, respectively (in the hypothetical case of structure variations occurring only at the quaternary and only at the tertiary level, R1 values would be 0 and 1, respectively).

An important point of this work is the quantification of asymmetry in homo‐oligomeric proteins; we used two semi‐quantitative parameters: the tertiary structure difference between subunits within the same quaternary assembly, measured by the RMSDs, and the presence of specific all‐or‐none differences (e.g. the presence of *cis* peptide bonds in some subunits of UPTase; or the distribution of ligands in partially saturated derivatives).

## Results

### D‐3‐phosphoglycerate dehydrogenase (PGDH) from *E. coli*

**Fig. 1:**
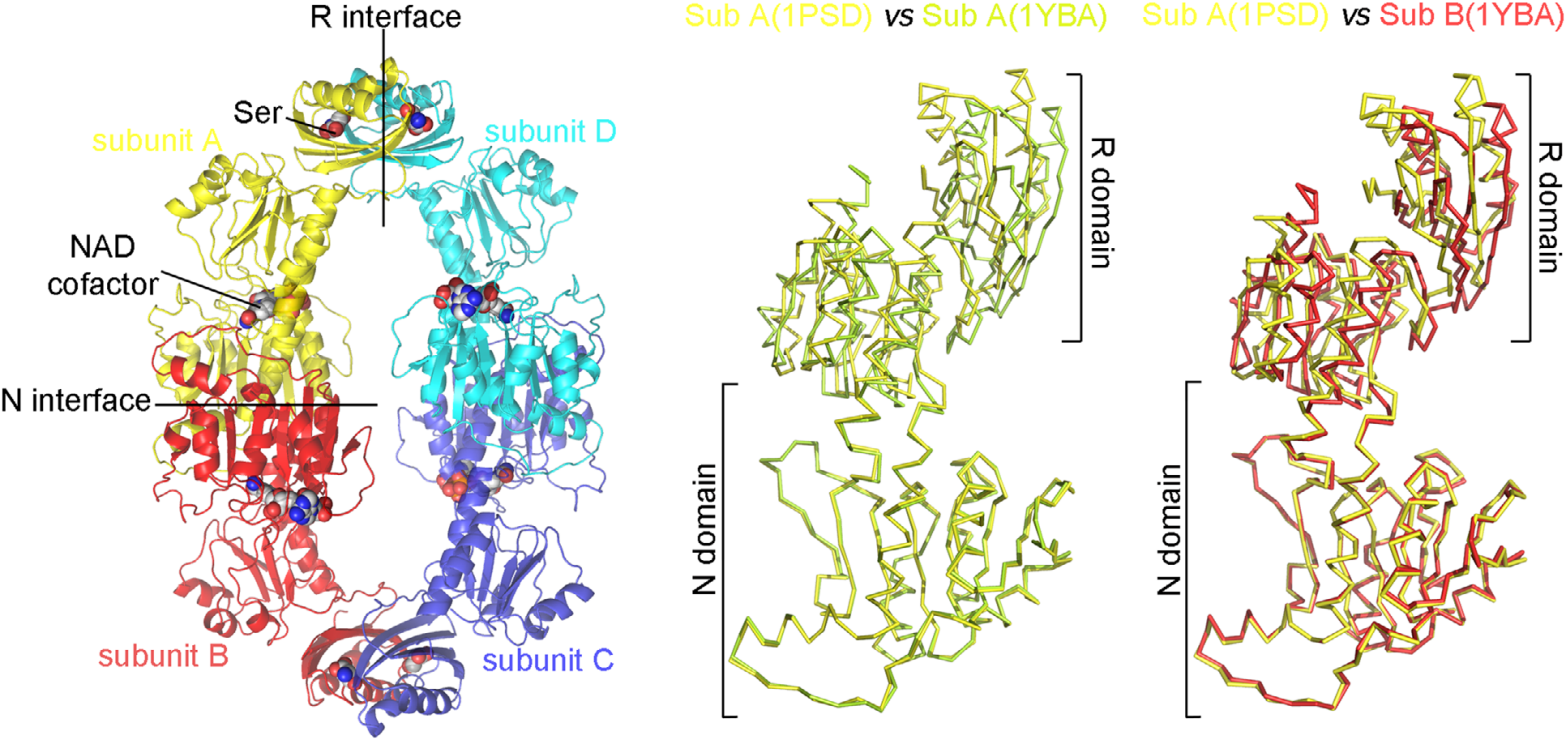
Structure of *E. coli* PGDH in the active and inhibited form. Left panel: tetrameric structure of phosphoglycerate dehydrogenase from *E. coli* (*Ec*PGDH) in the L‐Ser‐bound (i.e. inhibited) form (PDB ID: 1PSD; graphical elaboration was carried out with the software Chimera). The A, B, C and D subunits are shown as ribbon and coloured yellow, red, violet and cyan, respectively. The interfaces between R (regulatory) and N (NAD‐binding) domains are indicated at the top and on the left of the homotetramer structure, respectively. Equivalent R and N interfaces are located at the bottom and on the right corners of the homotetramer structure, respectively. The NAD substrate and L‐Ser inhibitor are shown as spheres and coloured by atom type: N, blue; O, red; C, white. Right panels: superposition of subunits A (light green) and B (red) of Ser‐free *Ec*PGDH (1YBA) with subunit A (yellow) of Ser‐bound *Ec*PGDH (1PSD), highlighting the tertiary structural changes and reorientation of the R domain with respect to the N domain between the two enzyme structures.

Bacterial D‐3‐phosphoglycerate dehydrogenase (PGDH) is the enzyme that catalyses the first step of the L‐serine biosynthesis pathway. The *E. coli* enzyme (*Ec*PGDH) is a homotetramer that presents negative homotropic cooperativity with respect to the phosphoglycerate substrate [19] and is heterotropically inhibited by L‐Ser, the final product of the pathway [20]. L‐Ser binds with a stoichiometry of one molecule per subunit (Fig. 1), causes a significant decrease of V_max_ and has essentially no effect on K_M_.

*E. coli* and related type II PGDHs are composed of four identical subunits (identified in the structure as A, B, C and D). Each subunit is made up of three domains (see Fig. 1): (i) the phosphoglycerate binding domain, called ‘S' (for substrate‐binding), which comprises two segments of the polypeptide chain, that is, the N terminus, and a segment located after the NADH‐binding domain; (ii) the NADH‐binding (or ‘N') domain; and (iii) the Ser‐binding regulatory (or ‘R') domain, at the C terminus [21]. In the tetramer, each subunit contacts two other subunits through two different types of isologous interfaces (*i.e*. symmetric interfaces comprising equivalent residues from different subunits [1]), which are contributed by the R and N domains of each subunit, respectively. Indeed, the *Ec*PGDH homotetramer has a rhomboidal overall shape and the structural arrangement of a dimer, or ring [35], of (two) dimers. The N‐N interfaces are N_A_‐N_B_ and N_C_‐N_D_, and the R interfaces are R_A_‐R_D_ and R_B_‐R_C_; in all interface names, subscript capital letters indicating the subunits participating in each interface, as in [21] (see Fig. 1). N‐N interfaces are larger than R‐R interfaces, and comprise two symmetric contact regions: one between residues 110–170 of one subunit and 266–300 of the partner subunit and the other between residues 266–300 of the first subunit and 110–170 of the partner subunit (interface contacts are listed in Table 1). R‐R interfaces involve contacts between residues 332–372 from both participating subunits. No contacts occur between subunits A and C or between subunits B and D.

**Table 1:** *Ec*PGDH contacts at subunit interfaces in experimentally determined 3D‐structures. Square brackets include residues contributed by a single subunit. X indicates that the contact is present in the crystallographic structure.

	PDB ID			
	1YBA		1PSD	
N interface	N_A_‐N_B_	N_B_‐N_A_	N_A_‐N_B_	N_B_‐N_A_
[111]–[125]	X	X	X	X
[111–114]–[148–150]	X	X	X	X
[115–122]–[115–122]	X	X	X	X
[134–140]–[286–292]	X	X	X	X
[172]–[172]	X	X	X	X
R interface	R_A_‐R_D_	R_D_‐R_A_	R_A_‐R_D_	R_D_‐R_A_
[335]–[371]	‐	‐	X	X
[346–365]–[346–366]	X	X	X	X
[366–368]–[370]	‐	‐	X	X
[366]–[372]	‐	‐	X	X
[369–372]–[369–372]	X	X	‐	‐

*Ec*PGDH has been suggested to operate according to the concerted model [21, 22]. However, this assignment is inconsistent with the protein structure and with the negative homotropic cooperativity with respect to both the phosphoglycerate substrate and the Ser inhibitor [19, 20]. To get insight in the cooperativity and regulation mechanisms of *Ec*PGDH, we analysed two structures: the active form, which is bound to four molecules of NAD^+^ and four molecules of the 2‐oxoglutaric (αKG), a nonphysiological substrate analogue (PDB ID: 1YBA) [24] and the inhibited form, which is bound to four molecules of NAD^+^ and four molecules of the L‐Ser inhibitor (PDB ID: 1PSD) [21].

According to the PDB, both the Ser‐free and Ser‐bound enzyme structures should present D2 dihedral symmetry, which indicates that the protein comprises four identical monomers and, therefore, has three symmetry axes. However, our current analysis indicates that while the Ser‐inhibited form is made of two identical but asymmetric dimers (AB and CD), and therefore presents C2 symmetry (only one symmetry axis), the active form presents considerable asymmetry (Table 2).

**Table 2:** Internal asymmetry and ligand‐induced structure changes in PGDH. The structural differences between the 1YBA and 1PSD monomers, dimers and tetramers are expressed by RMSD values (in Å) between the α carbons of 404 equivalent pairs of residues (residue range: 7–410) measured after rigid body alignment. Data in parentheses indicate that, due to internal symmetry in 1PSD, A = C and B = D.

PDB ID	Subunits: RMSD (Å)					
1YBA/1YBA	A/B: 0.51	A/C: 0.82	A/D: 1.23	B/C: 0.73	B/D: 1.02	C/D: 0.94
1PSD/1PSD	A/B: 1.46					
1YBA/1PSD	A/A: 2.03	B/A: 1.86	C/A: 1.76	D/A: 1.54		
1YBA/1PSD	A/B: 1.1	B/B: 0.92	C/B: 0.71	D/B: 0.98		
	Dimers: RMSD (Å)					
1YBA/1YBA	AB/CD: 1.14					
1PSD/1PSD	AB/CD: 0					
1YBA/1PSD	AB/AB: 1.95	DC/AB: 1.45				
	Tetramers: RMSD (Å)					
1YBA/1PSD	ABCD/ABA′B′: 3.36			BADC/ABA′B′: 3.03		

Table 2 reports data on the internal asymmetry of each structure and on the extent of ligand‐induced structure changes in *Ec*PGDH. In the 1PSD homotetramer, the two AB and CD homodimers are identical to each other. The asymmetry between the A and B monomers within the AB homodimer is significant (RMSD = 1.46 Å). In the 1YBA homotetramer, the AB and CD dimers have significant asymmetry (RMSD = 1.14 Å) and the four subunits are different from one another, with an average RMSD of 0.88 Å. The average structure difference between the subunits of 1PSD and 1YBA is 1.36 Å, a value that results from the relatively large structural difference between subunit A of 1PSD and all subunits of 1YBA (average RMSD = 1.80 Å) and the smaller difference between subunit B of 1PSD and all subunits of 1YBA (average RMSD = 0.93 Å). The smallest structure difference between the homotetramers of 1PSD and 1YBA is quantified by an RMSD = 3.03 Å. The ligand‐induced tertiary structure differences account for approximately 45% of the total (tertiary + quaternary) difference. In our previous analysis, we found that in typical MWC‐like proteins the tertiary structure change (estimated from the RMSD) accounts for approximately 25–30% of the total structure change, whereas this parameter is raised to 75% in typical KNF‐like proteins [5]; thus, PGDH does not belong to either set of typical proteins. However, given that the ligand‐induced tertiary structure change in PGDH has been described as a coordinated movement of the R and S domains relative to the N domain (Fig. 1) [21, 24], and that these domains form the intersubunit interfaces, it is no surprise that the quaternary structure change is relatively large. Indeed, a large tertiary structure change of this type in a subunit could not occur without perturbing the quaternary assembly. The data reported in Table 2 are incompatible with classical models of allostery or cooperativity, but we delay further analysis to the discussion section.

**Fig. 2:**
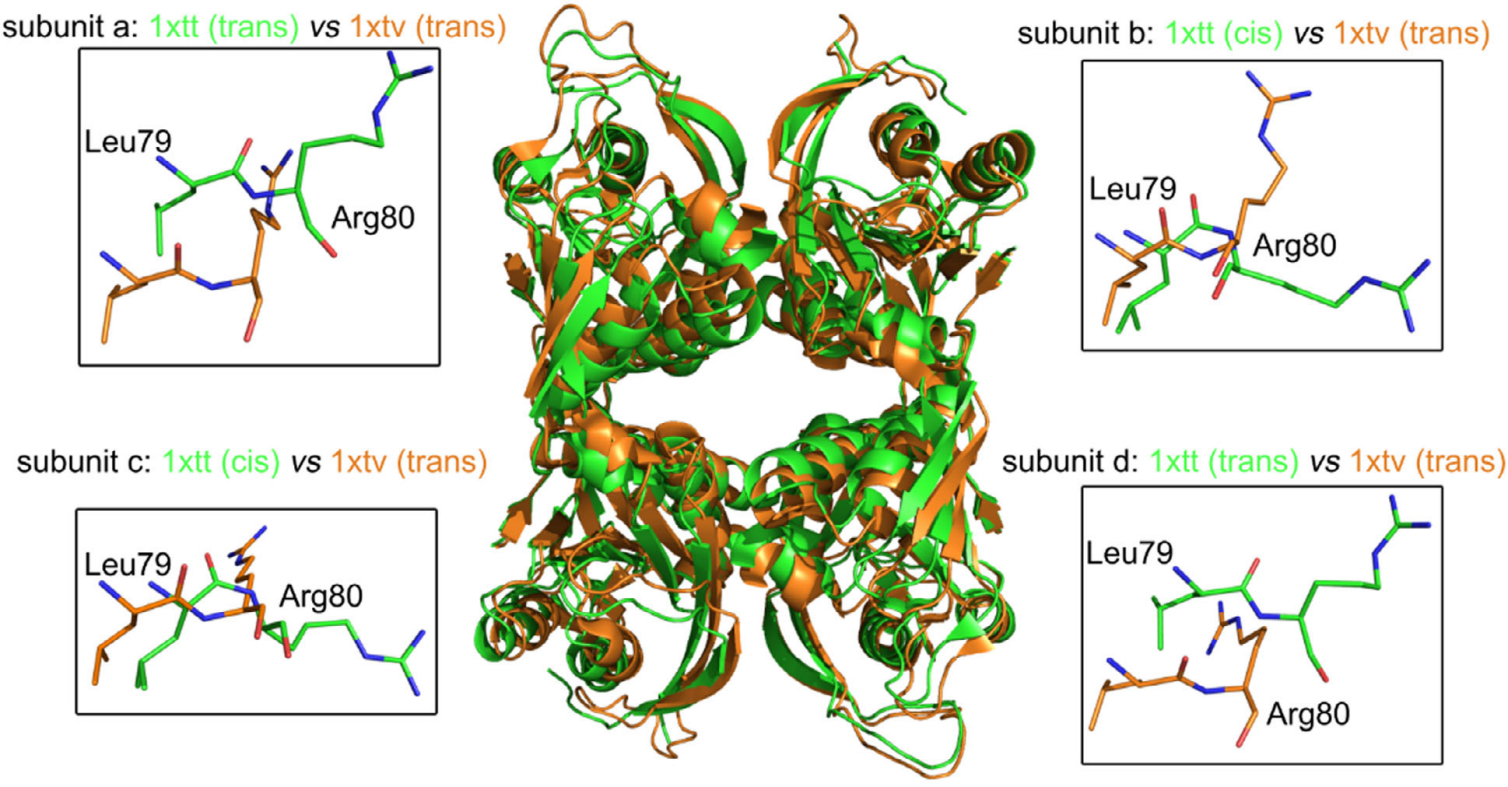
Quaternary and tertiary structure changes in *S. solfataricus* UTPase. Superposition of the whole uracil phosphoribosyltransferase from *S. solfataricus* (UTPase) tetramer with UMP bound to all four subunits (green cartoons; PDB ID: 1XTT) to the tetramer with UMP bound to two subunits only (orange cartoons; PDB ID: 1XTV). Graphical elaboration was carried out with the software Chimera. The four boxes highlight the tertiary conformational change relative to the Leu79‐Arg80 peptide that occurs in each subunit.

The catalytic mechanism of *Ec*PGDH and the inhibition by L‐Ser both present some important peculiarities, consistent with the structural asymmetry of the enzyme. The catalytic mechanism of PGDH is indicative of intramolecular functional heterogeneity, with two monomers in the tetramer accounting for 70% of the enzyme activity and the two other monomers for the remaining 30% [22]. Ser binding presents both positive and negative cooperativity. *Ec*PGDH comprises four identical Ser‐binding site located at the R interfaces (R_A_‐R_D_ and R_B_‐R_C_), each contributed by a.a. residues from the two interacting subunits. The distance between the two Ser‐binding sites across the R_A_‐R_D_ or R_B_‐R_C_ interfaces is <20 Å, whereas the distance between the two Ser‐binding sites across the N_A_‐N_B_ and N_C_‐N_D_ interfaces is >100 Å (Fig. 1). In 20 mm Tris buffer at pH = 7.5, the four intrinsic Ser dissociation constants were: K1 = 53.3 μm; K2 = 2.8 μm; K3 = 83.9 μm; K4 was too high to be determined (the standard errors on these constants are reported as vanishingly small). These values indicate positive cooperativity for the first two binding sites (K2 < < K1), with an estimated cooperative free energy of ΔG21 = 2.3 kcal/mol, and negative cooperativity for the subsequent site, with an estimated cooperative free energy of ΔG32 = −1.5 kcal/mol, as is clearly shown in Figs 2 and 3 of Grant et al. [20].

**Fig. 3:**
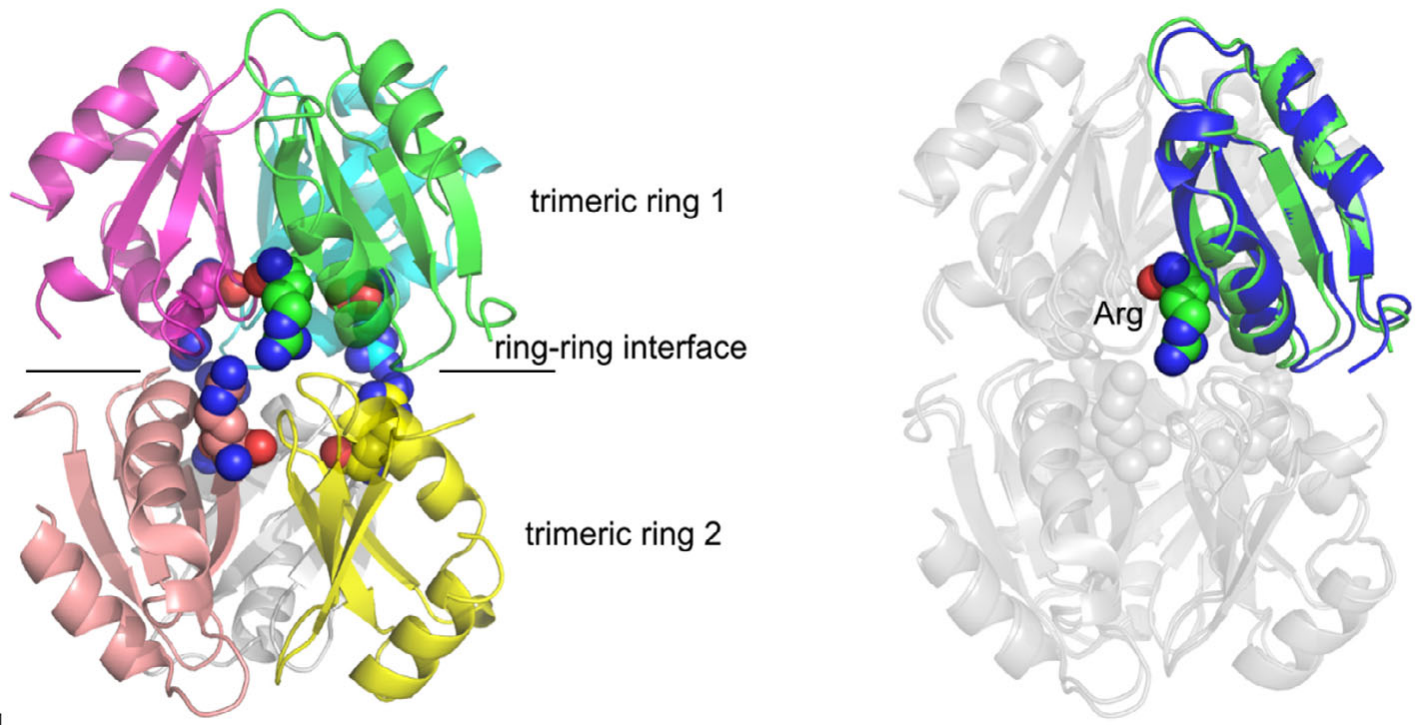
Arg‐bound Arg repressor from *E. coli*. Left panel: structure of the double ring structure of Arg‐bound repressor (PDB ID: 1XXA; graphical elaboration was carried out with the software Chimera). Arginine ligands are shown in spheres. Right panel: superposition of Arg‐bound and Arg‐free repressor (PDB ID: 1XXC; graphical elaboration was carried out with the software Chimera), two subunits belonging to the two hexamers are highlighted in green and blue cartoons.

Surprisingly, full inhibition of the enzymatic activity is already obtained when two (in Tris buffer) or three (in phosphate buffer) Ser molecules are bound; as a consequence inhibition presents positive homotropic cooperativity for Ser, and there is a discrepancy between the structural stoichiometry of the inhibitor (1 per subunit) and the functional stoichiometry (1 per dimer in Tris buffer, 1.5 per dimer in phosphate buffer) [20]. Given that the structure of the fully inhibited enzyme (PDB code 1PSD) is that of a symmetric dimer of asymmetric dimers, and given that perfect symmetry is consistent with positive cooperativity, whereas negative cooperativity implies asymmetry, it has been suggested that negative cooperativity for Ser occurs between sites that belong to the same R interface (i.e. R_A_‐R_D_ and R_B_‐R_C_) and positive cooperativity occurs between sites that do not belong to the same regulatory interface [23]. It would be of great interest to solve a structure containing only two molecules of bound Ser; this experiment should in principle be possible, given that binding of two Ser molecules gives rise to negative cooperativity, thus hampering binding of additional Ser molecules.

The study of the Ser‐binding sites, both in the active and inhibited structures is consistent with the above hypothesis. In the active form, which is bound to both NAD^+^ and αKG substrate analogue, but not to Ser (PDB ID: 1YBA), the two empty Ser‐binding sites across each R interface are asymmetric, since the two sites in the A and C subunits are in the open conformation and the two sites in the B and D subunits are in the closed conformation [24]. In spite of the asymmetry within each dimer, the tetramer is symmetric in terms of Ser‐binding sites, since each R interface comprises one open and one closed Ser‐binding site. It is plausible that the open/closed state of the Ser‐binding site correlates with the higher/lower catalytic activity of the subunit, even though we could not detect an obvious correlation between the open or closed structure of the Ser‐binding site and the structure of the catalytic site. In the presence of L‐Ser (PDB ID: 1PSD), all four sites are occupied by the inhibitor and assume closed conformation [21]; therefore, the symmetry of the R interfaces increases. However, the closed Ser‐bound sites in this structure are not perfectly identical; therefore some degree of asymmetry persists between the Ser‐bound sites in the A and B subunits.

### Uracil‐phosphoribosyl transferase from *S. solfataricus*

The enzyme uracil‐phosphoribosyl transferase (UPTase) from the archeon *S. solfataricus* is a homotetramer [25, 26]. Contrary to homologous enzymes from other sources, it presents no measurable ligand‐linked dissociation into dimers [26]. It has been characterized quite extensively, under the implicit assumption of a two‐state behaviour, but this assignment is doubtful. The enzyme is involved in the pyrimidine salvage pathway. It is activated by GTP and inhibited heterotropically by CTP and competitively by the UMP product [25, 26]. Each inhibitor has a weak effect by itself, but together they exert strong inhibition. UPTase requires two substrates, that is, uracil and phosphoribosyl pyrophosphate (PRPP), whose binding is mutually exclusive with respect to inhibitors. The dependence of V_max_ on the GTP activator is sigmoidal, with a Hill coefficient of 2.5. The affinity towards each substrate in the absence of the other, and towards GTP, CTP and UMP has been measured [25]. Binding of PRPP, GTP and CTP was found to be positively cooperative with Hill coefficients ranging between 1.5 and 4; UMP binds with small or absent cooperativity, whereas the uracil substrate presents negative cooperativity (Hill coefficient = 0.5). However, none of the ligands is able to saturate the enzyme, not even those that present the highest positive cooperativity, the observed maximum binding stoichiometry usually approaching 2 bound ligands per tetramer [25]. In the case of GTP, the largest functional effect is reached at 3 ligands per tetramer, but four ligands per tetramer are found in crystal soaking experiments. Thus UPTase, like PGDH, presents a discrepancy between the structural and functional stoichiometry of its substrate and effectors, and full inhibition is achieved before saturation of the enzyme with the inhibitor is reached. This behaviour suggests that intramolecular heterogeneity (*i.e*. negative cooperativity) is present. Indeed, it has been suggested that the binding of the first two ligand molecules is positively cooperative, but that of the last two is strongly negatively cooperative [25]. These results should be interpreted with great caution, because in some cases the reported Hill coefficients seem to exceed the number of bound ligands; hysteresis may be a possible reason of this observation, at least in the cases where the Hill coefficient was estimated from the rate of substrate transformation.

Three structures of the inhibited or partially inhibited enzyme are available: with UMP bound to all four subunits (1XTT); with both UMP and CTP bound to all four subunits (1XTU); and with UMP bound to only two subunits (1XTV) [26]. The fact that the structure of UPTase‐UMP_2_ (1XTV) could be solved is consistent with the marked negative cooperativity in binding of the UMP product to the active site of the enzyme. For the activated state of the enzyme, two structures are reported: one with four bound GTP molecules and four bound PRPP molecules (1VST); the other with four bound GTP molecules, two active sites occupied by PRPP and two by the hydrolysed products 5‐O‐phosphono‐alpha‐D‐ribofuranose (HSX), a ribose‐5‐phosphate analogue, and PP (3G6W), respectively [24]. The binding sites of the CTP inhibitor and GTP activator lie at the centre of the tetrameric structure and present partial overlap, thus simultaneous binding of these two effectors is prevented. The catalytic site accepts either U and PRPP or UMP and lies far away from the binding sites of GTP and CTP.

The quaternary arrangement of UPTase is that of a ring of two dimers, like that of PGDH. The tertiary structure of each subunit is made up of a core of five beta strands (β4, β5, β7, β8 and β9) flanked by three helices on one side (α1, α2 and α3) and two on the opposite side (α4 and α5). Two types of intersubunit interfaces are formed, both isologous: the largest one comprises parts of the N‐terminal region of each participating subunit, β‐strands β1, β2 and β3 and α‐helices α1, α2 and α3; the smallest one comprises parts of the C‐terminal region of each participating subunit and β‐strands β5 and β6 [27]. The four identical subunits, named A, B, C and D, are arranged in two AB and CD homodimers. Since subunits are not named according to the same topography in all UPTase structures deposited in the PDB, from now on we will refer to the subunits of all structures according to the nomenclature that they have in the activated structures (PDB ID: 1VST, 3G6W), as shown in Fig. 2.

The A subunit contacts the B via the large N‐terminal isologous interface, with a minor contribution of the C‐terminal regions of both subunits; the same applies to subunits C and D (Table 3). As in PGDH, the two AB and CD dimers contact each other via the isologous interfaces AD and BC, whereas no direct contact exists between the A and C subunits and the symmetric B and D subunits. However, subunits A and C can establish indirect contacts via the bound CTP or GTP effectors, and the same applies to subunits B and D. The interfaces are similar in all structures, but in the activated UPTase derivatives (PDB ID: 1VST and 3G6W), they are smaller than in the inhibited ones (PDB ID: 1XTU, 1XTT, 1XTV).

**Table 3:** UPTase interface contacts. X indicates presence of the contact in the structure.

PDB ID	1XTT		1XTU		1XTV		1VST		3G6W	
Ligands	UMP_4_		UMP_4_, CTP_4_		UMP_2_		GTP_4_, PRPP_4_		GTP_4_, PRPP_2_, (HSX + PP)_2_	
A/B and B/A contacts [residue numbers]	A‐B	A‐B	A‐B	B‐A	A‐B	B‐A	A‐B	B‐A	A‐B	B‐A
[8–10]–[41–49]	X	X	X	X	X	X	X	X	X	X
[12–16]–[65–70]	X	X	X	X	X	X	X	X	X	X
[20, 21]–[54–57]	X	X	X	X	X	X	X	X	X	X
[34]–[92]	‐	‐	‐	‐	X	‐	‐	‐	‐	‐
[38–42]–[38–42]	X	X	X	X	X	X	‐	‐	X	X
[56–60]–[203–208]	X	X	X	X	X	X	X	X	X	X
[92]–[92]	‐	‐	X	X	‐	‐	‐	‐	X	X
A/D and D/A contacts	A‐D	A‐D	A‐D	D‐A	A‐D	D‐A	A‐D	D‐A	A‐D	D‐A
[25–29]–[90–94]	X	X	‐	‐	‐	‐	‐	‐	‐	‐
[25–29]–[95–98]	‐	‐	X	X	X	X	X	X	X	X
[80]–[98–100]	X	X	‐	‐	‐	‐	‐	‐	‐	‐
[79–80]–[123–125]	‐	‐	X	X	X	X	‐	‐	‐	X
[98–100]–[215]	X	X	X	X	X	X	X	X	X	X
[122–123]–[122–123]	X	X	X	X	X	X	X	X	X	X
[125–127]–[212–216]	X	X	X	X	X	X	X	X	X	X

The analysis of UPTase interface contacts reveals some interesting, though not unexpected, details. All interfaces are, broadly speaking, isologous according to Monod's definition [1]. However, in some UPTase structures the interfaces are not perfectly symmetric, as expected based on the presence of negative cooperativity. As observed in the case of GPDH (see above), the highest interface symmetry occurs in the inhibited structures. In fact, in all the inhibited derivatives (PDB IDs: 1XTT, 1XTU and 1XTV) the A/B interface is essentially symmetric, whereas the A/D interface loses its symmetry when UPTase is bound to four UMP molecules (PDB ID: 1XTT).

The structure of the fully inhibited UPTase (UMP_4_CTP_4_; PDB code 1XTU) is highly symmetric, its four subunits being superimposable with RMSDs <<0.1 Å. As the degree of inhibition is progressively reduced (UMP_4_, PDB code 1XTT; UMP_2_ , PDB code 1XTV), the macromolecule becomes more asymmetric (Table 4). Not only, as in the case of PGDH, the noncontacting subunits (A and C, B and D) present smaller differences (estimated from the RMSDs) than each pair of contacting subunits, but some specific and highly characteristic differences appear, with the same pattern. Most notably in the 1XTT structure, the Leu79‐Arg80 peptide bond assumes cis configuration in the A and C subunits and trans configuration in B and D subunits (Fig. 2; see also Fig. 2B of Arent et al. [26]; note that in both the 1XTU and 1XTV structures all Leu79‐Arg80 peptide bonds have trans configuration). In the 1XTV structure, the asymmetry within the AB and CD dimers is also reflected by the two UMP molecules bound to subunits A and C, whereas subunits B and D are UMP‐free [27]. Thus, the core asymmetries observed in 1XTT and 1XTV are nonrandom, being internal to each of the AB and CD homodimers and resulting in tetramers that can be defined as quasi‐symmetric dimers of asymmetric homodimers. This condition is reminiscent of that observed in PGDH.

**Table 4:** Internal asymmetry of UPTase: RMSD between the α carbons measured after rigid body alignment of the isolated subunits or dimers.

Structure	A vs. B	A vs. C	A vs. D	B vs. C	B vs. D	C vs. D	AB vs. CD
1XTU	0.04 Å	0.04 Å	0.04 Å	0.05 Å	0.03 Å	0.06 Å	‐
1XTT	0.84 Å	0.28 Å	0.79 Å	1.47 Å	0.23 Å	0.81 Å	0.27 Å
1XTV	1.37 Å	0.52 Å	1.27 Å	1.31	0.49 Å	1.28 Å	0.59 Å
1VST	0	0	0	0	0	0	0
3G6W	0.62 Å	0.48 Å	0.72 Å	0.70 Å	0.43 Å	0.71 Å	0.51 Å

The case of the activated state of UPTase is somewhat similar. The structure 1VST, with four GTP and four PRPP bound has D2 symmetry, since all four subunits are identical to one another. Conversely, the structure 3G6W presents internal asymmetries and even though the RMSDs obtained from superposition of the subunits seem random, more specific features point to the similarity between the noncontacting subunits; indeed the two PRPP molecules are found in subunits A and C, whereas the two hydrolysed P‐ribose and PPi pairs are found in subunits B and D. Thus, as judging from the bound substrates or products, 3G6W behaves as a quasi‐symmetric dimer of asymmetric dimers.

To summarize, UPTase from *S. solfataricus* presents: (i) both positive and negative homotropic cooperativity; (ii) incomplete saturation with ligands; and (iii) an ordered asymmetry in the quaternary structures of at least some of its inhibited (1XTT and 1XTV) and activated (3G6W) states.

Comparison of the structures of inhibited and activated UPTase yields some interesting results (Table 5). The most obvious comparison is between structures 1XTU and 1VST: both are perfectly or almost perfectly symmetric D2 homotetramers and superposition of their constitutive subunits yields RMSD = 1.65 Å, whereas superposition of the tetramers yields RMSD = 3.14 Å. Thus, the ratio between tertiary and overall ligand‐induced structural changes is R1 = 0.52; not typical of either MWC‐ or KNF‐like proteins, but similar to the value obtained for PGDH. Quite surprisingly 1XTU (UMP_2_‐UPTase) is more similar to 1XTU (UMP_4_‐CTP_4_‐UPTase) than 1XTT (UMP_4_‐UPTase). The differences between the two activated structures 1VST and 3G6W are marked, in spite of the fact that these two derivatives differ only because one of them has four GTP and four PRPP bound (1VST) whereas the other has four GTP, two PRPP and two ribose‐5P analogues plus two PP bound (i.e. two molecules of a substrate and two analogues of a product).

**Table 5:** Ligand‐linked structure differences in UPTase: RMSDs between the α carbons measured after rigid body alignment of the tetramers or the isolated subunits. Values in parentheses indicate that, due to internal symmetry, subunits A and B of structure 1VST are identical.

Structures compared	Tetramers	Subunit A vs. A	Subunit A vs. B	Subunit B vs. A	Subunit B vs. B
1XTT vs. 1XTV	1.86 Å	0.61 Å	1.50 Å	1.52 Å	1.15 Å
1XTT vs. 1XTU	1.58 Å	0.68 Å	0.67 Å	1.57 Å	1.57 Å
1XTV vs. 1XTU	0.7 Å	0.37 Å	0.38 Å	1.48 Å	1.48 Å
1VST vs. 3G6W	1.31 Å	1.48 Å	0.50 Å	(1.48 Å)	(0.50 Å)
1XTU vs. 1VST	3.14 Å	1.65 Å	1.65 Å	(1.65 Å)	(1.65 Å)
1VST vs. 1XTT		1.55 Å	1.02 Å	1.54 Å	0.99 Å
1VST vs. 1XTV		1.58 Å	0.86 Å	1.49 Å	0.90 Å
1XTU vs. 3G6W		0.76 Å	0.93 Å	0.83 Å	0.99 Å
1XTT vs. 3G6W		0.52 Å	0.81 Å	1.42 Å	0.6 Å

The apparently paradoxical finding of tertiary differences greater than the overall ones is explained by the fact that since the macromolecule presents a nonrandom internal asymmetry, some superpositions of single subunits compare subunits that would never be superimposed in the overall superposition of whole tetramers.

The structural features of the different derivatives of UPTase are difficult to reconcile with a two‐state model (MWC or VS), both because of the internal asymmetries and because of the apparent plasticity of the quaternary structure, most probably indicative of an extended conformational ensemble (see the Discussion section).

### Arg repressor (*E. coli*)

The Arg repressor of *E. coli* has a six‐mer structure, with 6 Arg binding sites located at intersubunit interfaces [28, 29]; three subunits contribute residues to each Arg binding site (Fig. 3). The overall quaternary structure may be described as two trimeric rings stacked one on top of the other; thus, the protein has the symmetries of a dimer of rings [35]. As characteristic of this type of assembly, the six‐mer presents both isologous and heterologous interfaces, and its dissociation proceeds via separation into two trimers (which only have heterologous interfaces). Although the published structures are very similar both in the Arg‐free and Arg‐liganded states, with minimal quaternary structure changes, the crystals of the Arg‐free protein crack when exposed to Arg [29]. This phenomenon is an indicator of a quaternary structure change, however small; in this case, it is likely due to the fact that the binding site of Arg lies at the interface of three monomers, and its binding perturbs the interface contacts between subunits; the quaternary conformation change, though small, is evidently sufficient to disrupt the packing of the molecules in the crystal. As typical of oligomeric proteins presenting the ligand binding sites at intersubunit interfaces, Arg binding is coupled to strong quaternary enhancement (i.e. the Arg‐liganded six‐mer structure has less tendency to dissociate into trimers than the Arg‐free oligomer). The protein seems to present a significant negative cooperativity for Arg, the first ligand binding with 100‐fold higher affinity than the following five [29].

The structure parameters are as follows: the Arg‐free (PDB code: 1XXC) and Arg‐bound (PDB code: 1XXA) are both slightly asymmetric homohexamers; superposition of the two hexamers yields RMSD = 0,88 Å. The average RMSDs for tertiary superpositions are approx. the same for 1XXA, 1XXC and the subunits of 1XXA vs. those of 1XXC, circa 0.70 Å. Thus tertiary structure changes account for 80% of the total structure change. This value is fully consistent with those found for typical sequential proteins [17].

Arg repressor has small intramolecular asymmetries, which do not appear to follow any particular order; in particular, the two rings are not exactly symmetrical in either of the two structures we analysed; moreover, the Arg‐induced quaternary structure change is very small (Table 6). Because of these features, it seems likely that regulatory effects may be played at the tertiary structure level.

**Table 6:** Internal asymmetry of Arg repressor: RMSDs between the α carbons measured after rigid body alignment of the isolated subunits, trimers and dimers. Values in parentheses are duplicates; a dash indicates that the structures to be superimposed are one and the same.

	1XXC	1XXA	1XXC vs. 1XXA	1XXA vs. 1XXC
Subunit vs. subunit	0.46–0.65 Å	0.41–1.1 Å	0.6–1.1 Å	(0.6–1.1 Å)
Trimer ABC vs. trimer ABC	‐	‐	0.78 Å	(0.78 Å)
Trimer DFE vs. trimer DFE	‐	‐		
Trimer ABC vs. trimer DFE	0.57 Å			1.01 Å
Hexamer vs. hexamer	‐	‐	0.88 Å	(0.88 Å)

### Comparisons

Given that our inclusion criteria were stringent and only three proteins from reference [7] matched them, it is of some interest to compare the results we obtained on PGDH and UPTase with a similar analysis carried out on other homotetramers.

**Fig. 4:**
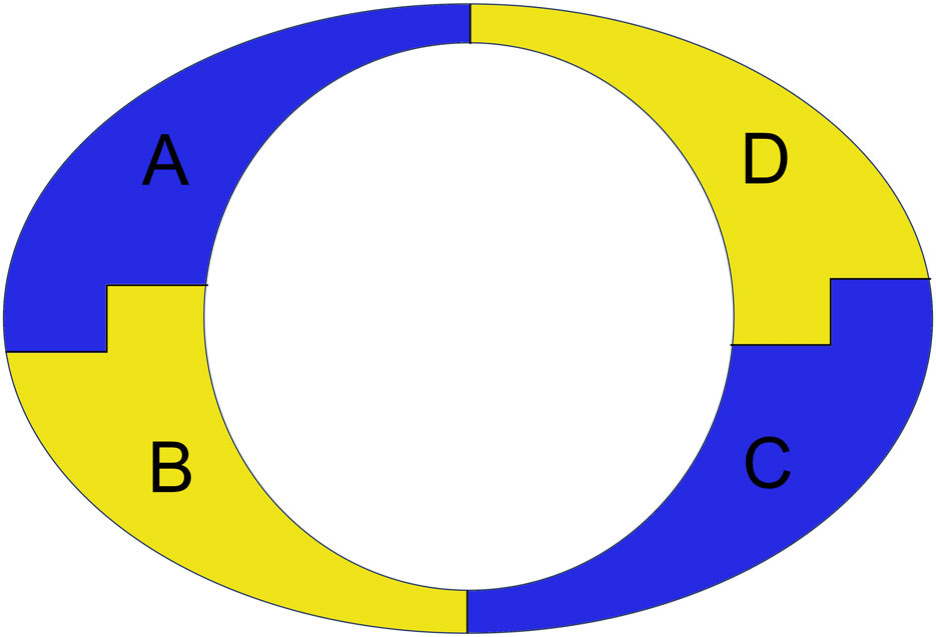
Schematic representation of the ordered asymmetry observed in the fully L‐Ser‐inhibited *Ec*PGDH (1PSD), in the incompletely inhibited UPTase (1XTT, 1XTV) and in the activated UPTase (3G6W). The phosphoglycerate dehydrogenase from *E. coli* (*Ec*PGDH) and uracil phosphoribosyltransferase from *S. solfataricus* (UPTase) homotetramers are made of two identical dimers (AB and CD). The dimers are internally asymmetric, because of tertiary structure differences between the constituting monomers. All interfaces are isologous, the symmetric AB and CD being larger than the symmetric AD and BC. No contact exists between subunits A and C and between subunits B and D. The subunits that do not have direct contacts with each other have very similar or identical tertiary structure and are depicted in the same colour, whereas the tertiary structure of each subunit differs from that of contacting subunits. In spite of the asymmetries between the subunits, these tetramers are classified in the PDB as presenting D2 symmetry, indicating that they have three twofold axes. However, D2 symmetry requires that all subunits are identical, whereas the A and C subunits are not identical to the B and D subunits. Conversely, the tetramers have C2 symmetry, thanks to the presence of one symmetry axis that allows an identical molecule to be obtained following a 180° rotation around this axis (in this example, the 180° rotation around the C2 axis places the CD monomer in the position previously occupied by the identical AB monomer, and *vice versa*). These tetramers present C2 symmetry from a functional viewpoint as well, since binding of ligands occurs with higher affinity, and possibly with positive cooperativity, to subunits (that are not contact with each other: that are symmetric with respect to a C2 axis, suggesting a quaternary, concerted conformational change, whereas negative cooperativity occurs for the third and fourth ligands, which bind to the two remaining, C2‐symmetry related subunits).

Fructose bis‐phosphatase (FBPase) is a positively cooperative enzyme, with the structure of a homotetramer, heterotropically inhibited by AMP and ATP [36]. Positively cooperative enzymes may operate via every conceivable cooperativity model, either requiring perfect symmetry (MWC and its variants) or asymmetry (KNF); thus, no *a priori* prediction can be made on their structure. FBPase is supposed to operate via a MWC reaction scheme because its quaternary structure is a dimer of dimers with isologous interfaces, and the ligand‐induced structure change involves a rotation of one dimer with respect to the other, reminiscent of haemoglobin, which is a certain example of MWC. However, the allosteric equilibrium between the T and R state was not demonstrated; thus, the attribution of a MWC‐like reaction mechanism should be considered provisional. Indeed, as shown in Table 7, below, the inhibited, putative T state of the enzyme (structures 1EYJ and 1EYK) presents a significant internal asymmetry, reminiscent of the one illustrated in Fig. 4, whereas the putative R state (structure 1EYI) is perfectly symmetric [36]. We also remark that the FBPase tetramer forms a compact structure, in which every subunit contacts all other three; thus, in spite of the difference in the surface area buried in the different intersubunit interfaces, it does not possess the ring‐like quaternary structure of PGDH and UPTase.

**Table 7:** Structure differences measured from the RMSDs in selected protein. The table reports the results of pairwise comparison of the subunits; where subunits are not specifically named the observed asymmetry was disordered and a range of RMSDs is provided.

Protein	Comparison	Comparison	Comparison
FBPase	1EYJ (T state) A vs. B: 1.43 Å	1EYK (T state) A vs. B: 1.38 Å	1EYI (R state): A vs. B: 0
D‐LacDH	6ABJ (substrate‐free) A vs. B: 0.31 Å	5Z20 (substrate‐bound) overall 0.59–1.09 Å	
DAHPS	1KFL (inhibited): overall: 0.17–0.34 Å	1GG1 (substrate‐bound): overall 0.26–0.3 Å	
G3PDH	1J0X: overall: 0.24–0.42 Å		

Bacterial D‐lactate dehydrogenases (D‐LacDH) are D2‐homotetramers and present negative cooperativity. In the substrate‐free state, the enzyme from *Ps. aeruginosa* (structure 6ABJ) is almost symmetric, with a hint of the condition illustrated in Fig. 4, whereas in the substrate‐bound state (structure 5Z20), it presents a disordered internal asymmetry in which no clear pattern can be detected (Table 7). The enzyme was assigned (we believe correctly) a KNF reaction mechanism, which presumes asymmetry regulated by pairwise intersubunit interactions and does not require any special order at the quaternary structure level [15]. The homotetramer has a compact structure, but each subunit contacts only two others, in this resembling PGDH and UPTase. D‐LacDH failed inclusion in the main analysis because it has no known heterotropic effectors.

3‐deoxy‐D‐arabino‐heptulosonate‐7‐phosphate synthase (DAHPS) is a noncooperative D2‐homotetramer with a compact structure in which each subunit contacts all other three. The enzyme catalyses the initial step in the biosynthesis of aromatic amino acids, and there are three isoforms one for each aromatic amino acid and inhibited by the terminal product of the biosynthetic pathway. The isoform that participates in the biosynthesis of L‐Phe (and is inhibited by this amino acid) is the best characterized. The structures of the inhibited enzyme (bound to L‐Phe and P‐enol piruvate, PDB ID: 1KFL) and of the enzyme bound to the substrate analogue P‐glycolate (PDB ID: 1GG1) both present minor internal asymmetries without any particular order. Inhibition by L‐Phe is suggested to operate at the tertiary structure level [37, 38].

Glyceraldehyde 3 phosphate dehydrogenase (G3PDH; PDB ID: 1J0X) is a classical example of a negatively cooperative enzyme. The mammalian enzyme (from rabbit) is a compact homotetramer with D2 symmetry, in which each subunit contacts all other three [16]. The subunits present minor asymmetries, with no obvious distribution pattern. G3PDH is regulated by several covalent, but potentially reversible, modifications, but no noncovalent heterotropic ligand, thus failing the inclusion criteria for this study.

## Discussion

Regulation of cell functions is key to physiology, and dysregulation is almost always a cause of disease. Regulation of protein, and thus of cell, function is achieved via structure changes induced by the binding of specific ligands; this type of regulation is usually called allosteric, but, as we discussed in a recent paper [18], the common usage of this term lacks precision. In this paper we compare the structural and functional features of heterotropic regulation in proteins that present negative homotropic cooperativity. Both negative homotropic cooperativity and heterotropic regulation are frequently encountered, but their association is uncommon: heterotropic regulation being usually associated with positive or absent cooperativity.

In this study, we address the structural basis of heterotropic regulation in negatively cooperative proteins and we find that, in spite of the very low number of proteins we were able to select, some features stand out: (i) the three proteins studied present the structure of rings; ArgR is a dimer of rings, PGDH and UPTase are rings of dimers [35]; (ii) as characteristic of this type of arrangement, some subunits do not contact each other (unless via effector molecules); thus, they cannot directly exchange information as hypothesized by the classical KNF model; (iii) in all three proteins the structural stoichiometry is one molecule of the effector *per* subunit; (iv) the binding site of the effector lies in the proximity of the intersubunit interfaces. (v) Due to negative cooperativity of effector binding, full saturation may not be achieved or may require very high, possibly non physiological concentrations of the effector(s); accordingly, activation/inhibition may not require that all subunits are bound to the effector, that is, the functional stoichiometry of effector binding may be lower than one *per* subunit. The most obvious example is provided by PGDH, which has four binding sites for the inhibitor Ser (one *per* subunit) but is fully inhibited when two molecules of Ser are bound (functional stoichiometry being two *per* tetramer). This property has the paradoxical effect that if one studies the binding of Ser to PGDH one finds that the tetramer binds four molecules of Ser with negative cooperativity, whereas if one studies the inhibition of enzymatic activity by Ser one finds that the tetramer binds two molecules of Ser with positive cooperativity (the binding of the last two molecules being functionally silent). The cases of Arg repressor and UPTase are remarkably similar since also in these cases effector binding is negatively cooperative and full inhibition seems not to require full saturation of the effector binding sites. The above described properties imply a reaction mechanism that involves a quaternary structural transition, albeit not necessarily one requiring the ligation‐independent between two different conformations.

If we limit our comparison with the two homotetramers, PGDH and UPTase, the structural and functional similarities are even more striking, in spite of the two proteins being completely unrelated. Indeed, in both of them, internal asymmetry of the subunits is evident, as required by the negative cooperativity, and also ordered, according to the pattern shown in Fig. 4, whereby noncontacting subunits are more similar or identical than reciprocally contacting subunits. In UPTase structures 1XTT and 3G6W, asymmetries are also apparent at the AD interface, where we observe that some contacts that should be isologous appear only once instead of twice. As a general rule, the more relevant interface changes occur at the ‘small’ interfaces (AD and BC). Both enzymes present positive and negative cooperativity, and it seems plausible that negative cooperativity may be associated with asymmetry and may occur between contacting subunits, possibly via the small interface, whereas positive cooperativity would require symmetry and would occur between the noncontacting subunits AC and/or BD (see Fig. 4), as a consequence of a quaternary structure rearrangement.

It is difficult to generalize the findings described in this work; surely many proteins present ring‐like symmetry [35] and many tetramers present C2 symmetry. However, the proteins selected for this comparative study present a remarkable association of structural and functional features, not easily found together in the majority of allosteric proteins, for example, the discrepancy between the number of effector binding sites and the number of bound effectors required to achieve maximal inhibition, or the alternance of positive and negative cooperativity.

The structural properties and functional behaviour of the proteins analysed in this study are incompatible with either concerted or sequential models, but may present features of both. The main discrepancy between the properties of these proteins and the two‐state allosteric model of Viratelle and Seydoux, which requires C2 symmetry [6], lies in the fact that these proteins seem to have more than two energy states, and UPTase clearly has more than two quaternary structures; thus, the functional properties of these proteins may be conveniently interpreted as resulting from a conformational and energy landscape with multiple minima (i.e. a conformational ensemble), of which the MWC and KNF models may be viewed as two special cases. Unfortunately, our type of structure analysis can only be applied to the structures that have actually been solved, which are probably only a fraction of those effectively accessible to these proteins. Moreover, the functional data are clearly insufficient to assess the binding free energies of the different structures. We also remark that our structural analysis is difficult to reconcile with the KNF model because cooperative interactions seem to occur between noncontacting subunits. Indeed, it seems to us that a plausible description of the proteins here analysed should be looked for in the ‘nesting’ concept developed by J. Wyman [39]. Nesting implies a hierarchy of cooperative and regulatory interactions by which a cooperative oligomer is embedded in a larger and more cooperative superstructure and has been applied to the description of several biochemical systems, including haemocyanins [40] and chaperonins [41]. Nesting can be reconciled with the idea of a conformational ensemble, with the proviso that each hierarchic level of structural organization has its own energy landscape. In the case of PGDH and UPTase. the smaller functional unit would be the asymmetric homodimer, presenting negative cooperativity, whereas the larger assembly would be the tetramer, looked at not as the assembly of four identical subunits, but as the assembly of two (asymmetric) homodimers, presenting positive cooperativity. In the case of the Arg repressor. it seems appropriate to identify the smaller functional structure in the trimeric ring, and the superstructure in the dimer of rings. The lower level structural assembly (either a dimer or a trimer) might operate according to a KNF‐like sequential behaviour, whereas the higher level assembly (either a tetramer or a six‐mer) would possibly operate according to a MWC‐like two‐state behaviour.

What would be the physiological and evolutionary advantage of such a complex behaviour? As Wyman himself pointed out long ago, ‘An advantage of this arrangement in terms of function … may be extension of the range of cooperative binding, enabling the macromolecule to operate with different cooperativities in different regions of ligand activity’ [40]. While positive cooperativity in an enzyme buffers. the concentration of the substrate by maximizing the increase in consumption as a response to an increase in availability, negative cooperativity buffers the velocity of the reaction and thereby helps maintain a constant metabolic flow [42]. Thus it is conceivable that the same enzyme might be subject to two opposite evolutionary pressures and that different ranges of substrate concentrations may require different functional responses and catalytic efficiencies. This extreme type of adaptation would require that both positive and negative cooperativity may be obtained and selected via heterotropic regulation.

## Conflict of interest

The authors declare no conflicts of interest.

## Author contributions

A.B. conceived and designed the study. V.M. and PDM designed and implemented the program Face2Face for the analysis of interfaces. V.M. and F. Angelucci supervised the structure analyses. F. Arnesano and GMP carried out the structural superpositions.

## Data Availability

Structure data were obtained from the PDB; the results of the structure analysis carried out are entirely reported in the Tables; the programs used are described in the methods; the server face2face is available at the link provided above. Further queries can be addressed to the corresponding author.
